# Proteome-Wide Analysis of Lysine 2-Hydroxyisobutyrylation in the Phytopathogenic Fungus *Botrytis cinerea*

**DOI:** 10.3389/fmicb.2020.585614

**Published:** 2020-11-27

**Authors:** Yang Xu, Xiaoxia Li, Wenxing Liang, Mengjie Liu

**Affiliations:** Key Laboratory of Integrated Crop Pest Management of Shandong Province, College of Plant Health and Medicine, Qingdao Agricultural University, Qingdao, China

**Keywords:** proteome, lysine 2-hydroxyisobutyrylation, bioinformatics analysis, *Botrytis cinerea*, pathogenicity

## Abstract

Posttranslational modifications (PTMs) of the whole proteome have become a hot topic in the research field of epigenetics, and an increasing number of PTM types have been identified and shown to play significant roles in different cellular processes. Protein lysine 2-hydroxyisobutyrylation (K_*hib*_) is a newly detected PTM, and the 2-hydroxyisobutyrylome has been identified in several species. *Botrytis cinerea* is recognized as one of the most destructive pathogens due to its broad host distribution and very large economic losses; thus the many aspects of its pathogenesis have been continuously studied. However, distribution and function of K_*hib*_ in this phytopathogenic fungus are not clear. In this study, a proteome-wide analysis of K_*hib*_ in *B. cinerea* was performed, and 5,398 K_*hib*_ sites on 1,181 proteins were identified. Bioinformatics analysis showed that the 2-hydroxyisobutyrylome in *B. cinerea* contains both conserved proteins and novel proteins when compared with K_*hib*_ proteins in other species. Functional classification, functional enrichment and protein interaction network analyses showed that K_*hib*_ proteins are widely distributed in cellular compartments and involved in diverse cellular processes. Significantly, 37 proteins involved in different aspects of regulating the pathogenicity of *B. cinerea* were detected as K_*hib*_ proteins. Our results provide a comprehensive view of the 2-hydroxyisobutyrylome and lay a foundation for further studying the regulatory mechanism of K_*hib*_ in both *B. cinerea* and other plant pathogens.

## Introduction

Protein posttranslational modifications (PTMs) are important regulatory mechanisms in all living cells and involved in almost all aspects of cellular processes. To date, more than 400 PTMs have been identified from eukaryotes and prokaryotes, and newly discovered PTMs are regularly being reported; PTMs greatly enrich the functions of proteins by affecting protein activity, stability, localization, and interactions (Hart and Ball, 2013; Vu et al., 2018). Major types of PTMs, such as phosphorylation, ubiquitination, glycosylation, acylation, lipidation, thiolation, and oxidation, have been well studied, and these PTMs can finely regulate cellular responses to the slightest changes in the environment through single PTM regulatory or PTMs crosstalk (Walsh et al., 2005; Vu et al., 2018; Macek et al., 2019).

In the peptide chain, multiple amino acid residues can be covalently modified by different groups (Macek et al., 2019). Protein acylation mainly occurs on lysine residues, which are modified by short-chain fatty acids donated by their corresponding acyl-coenzyme A (CoA) groups (Huang et al., 2018). The most studied protein acylation type is histone acetylation, which was discovered more than 50 years ago (Allfrey et al., 1964). Acetylation was identified in non-histone proteins and has been shown to also play significant roles in protein function (Dancy and Cole, 2015). Apart from acetyl groups, a variety of short-chain fatty acid groups have been discovered on lysine residues of mature proteins, including propionylation (K_*pr*_), butyrylation (K_*bu*_), crotonylation (K_*cr*_), 2-hydroxyisobutyrylation (K_*hib*_), malonylation (K_*mal*_), and succinylation (K_*su*_) (Walsh et al., 2005; Chen et al., 2007; Zhang et al., 2011; Huang et al., 2014; Zhao and Garcia, 2015).

K_*hib*_ is a newly identified protein posttranslational lysine acylation modification that is derived from 2-hydroxyisobutyryl-CoA (Dai et al., 2014). In humans and mice, compared to histone lysine acetylation (K_*ac*_) and K_*cr*_, histone K_*hib*_ showed a unique chemical structure and distinct genomic distribution. Moreover, in male germ cells, the 2-hydroxyisobutyrylation of the 8th site lysine residue in histone 4 (H4K8_*h*__*ib*_) is associated with active gene transcription in both meiotic and postmeiotic cells. Thus, K_*hib*_ is considered to be a new histone marker and plays a unique function (Dai et al., 2014; Huang et al., 2018). The yeast histone acetyltransferase complex NuA4 and human acetyltransferase Tip60 have been shown to function as enzymes to catalyze K_*hib*_, while histone deacetylase 2 (HDAC2) and histone deacetylase 3 (HDAC3) function as the major enzymes to remove 2-hydroxyisobutyryl from K_*hib*_ in mammalian cells (Huang et al., 2018), suggesting that there may be an internal relation between K_*ac*_ and K_*hib*_. In recent years, K_*hib*_ has been detected and characterized in both histone and non-histone proteins in several species using newly developed modern techniques in molecular biology and mass spectrometry (Huang et al., 2017; Meng et al., 2017; Yu et al., 2017; Dong et al., 2018; Yin et al., 2019). For example, a total of 6,548 unique K_*hib*_ sites on 1,725 proteins were identified in human cells (Huang et al., 2018). In yeast, a total of 1,458 K_*hib*_ sites on 369 proteins were identified, among which 206 proteins were also modified by both acetylation and succinylation (Huang et al., 2017). A total of 9,916 K_*hib*_ sites on 2,512 proteins and 11,976 K_*hib*_ sites on 3,001 proteins were identified in developing rice seeds and *Physcomitrella patens*, respectively, showing a large 2-hydroxyisobutyrylome in plants (Meng et al., 2017; Yu et al., 2017). Bioinformatics analyses showed that the identified K_*hib*_ proteins were closely associated with a wide variety of cellular processes, such as protein synthesis and processing, protein degradation, translation, and energy metabolism (Huang et al., 2017, 2018; Yin et al., 2019), indicating that K_*hib*_ plays a broad and significant role in cellular processes.

*Botrytis cinerea*, the pathogen of gray mold, is considered to be a broad generalist pathogen due to its broad host distribution from bryophytes to eudicots, and can cause severe pre- and post-harvest losses in crops (Dean et al., 2012; Soltis et al., 2019). In addition, *B. cinerea* is considered a typical necrotroph, and its growth and pathogenic mechanisms have been well studied. However, until now, only a few modification-specific *B. cinerea* proteomics studies have been reported, including studies on the phosphoproteome and acetylome of *B. cinerea* (Liñeiro et al., 2016; Lv et al., 2016). To understand K_*hib*_ modification and its function in *B. cinerea*, the 2-hydroxyisobutyrylome of the mycelium was investigated using proteome-wide analysis, and a total of 5,398 K_*hib*_ sites on 1,181 proteins were identified. Subsequently, characteristics of K_*hib*_ site motifs, the conservation of the K_*hib*_ proteins compared to other species, the functional classification and enrichment, and the protein–protein interaction (PPI) network were analyzed. Finally, the reported pathogenicity-related proteins in the identified K_*hib*_ proteins were summarized. Our results show that K_*hib*_ is an important PTM and is involved in the regulation of various cellular processes in the phytopathogenic fungus *B. cinerea*.

## Materials and Methods

### Fungal Strain and Culture

The *Botrytis cinerea* model strain B05.10 was used in this study. Spores or mycelium of *B. cinerea* were inoculated on potato dextrose agar medium (PDA) and cultured in incubator under the condition of dark and 25°C for 5 days. Conidia were collected from the plate using sterile distilled water and then counted using blood counting chamber. Conidia with a final concentration of 5 × 10^4^ cfu were incubated in yeast extract peptone dextrose medium (YEPD) and cultured in shaker under the condition of 25°C and 150 rpm for 16 h. Mycelium, the vegetative body and the main infection structure of *B. cinerea*, was harvested by filtering with sterile gauze, immediately frozen in liquid nitrogen and then stored at −80°C.

### Total Protein Extraction

Total protein extraction from the mycelium according to previous methods (Baker and Panisko, 2011) with some modifications. Briefly, accurately weigh 300 mg mycelium and grind it to powder in liquid nitrogen. The cell powder was transferred into a 2 ml centrifuge tube containing 1 ml lysis buffer (1 M sucrose, 0.5 M Tris-HCl (pH8.0), 0.1 M KCl, 50 mM ascorbic acid, 1% NP40, 1% sodium deoxycholate (NaDOC), 10 mM ethylenediamine tetraacetic acid (EDTA), 10 mM dithiothreitol (DTT), 3 μM trichostatin A (TSA), 50 mM nicotinamide and 1% protease inhibitor cocktail), in which the TSA and nicotinamide were used as de-2-hydroxyisobutyrylase inhibitors to maintained the modification level of proteins extracted from cells. The powder was dissolved by sonication on ice followed by keeping on ice for 10 min. Add 1 ml of Tris-saturated phenol into a centrifuge tube, well mixed and leave on ice for another 10 min. The upper phenol phase (about 800 μl) was transferred to a new 10 ml centrifuge tube after centrifuged under 16,000 *g* at 4°C for 10 min, followed by adding 4 ml −20°C precooled 0.1 M ammonium acetate dissolved in pure methanol and stayed at −20°C overnight to precipitate protein. After centrifugation under 16,000 *g* at 4°C for 10 min and discarding the supernatant, precipitate was successively washed once with −20°C precooled methanol and twice with −20°C precooled acetone. Then, the remaining precipitate was moderately air-dried and resolved in 0.8 ml protein lysis buffer (8 M urea, 50 mM Tris-HCl (pH8.0), 1% NP40, 1% NaDOC, 10 mM EDTA, 5 mM DTT, 3 μM TSA, 50 mM nicotinamide and 1% protease inhibitor cocktail) by sonication on ice. Finally, the supernatant was transferred into a new 1.5 ml tube after centrifugation under 20,000 *g* at 4°C for 10 min and the protein concentration was determined with 2-D Quant kit (GE Healthcare) according to manufacturer’s instructions.

### Protein Reduction, Alkylation, and Trypsin Digestion

DTT was added to 3 μg protein in solution to a final concentration of 10 mM and incubated for 1 h at 37°C for reduction reaction, followed by alkylated with 30 mM iodoacetamide (IAM) for 45 min at room temperature in darkness. The solution was stayed at −20°C overnight to precipitate protein by adding four times volume −20°C precooled acetone. After centrifugation under 20,000 *g* at 4°C for 10 min and discarding the supernatant, precipitate was washed twice with −20°C precooled acetone. The remaining precipitate was moderately air-dried and resolved in 0.1 M TEAB by sonication on ice. For digestion, 60 μg trypsin was added to the protein solution, kept at 37°C overnight, and then reaction was stopped by adding 1% trifluoroacetic acid (TFA), followed by desalination using C18 SPE column (5 μm particles, 4.6 mm ID, 250 mm length). Finally, peptides were dried by vacuum centrifuging.

### K_*hib*_ Peptides Affinity Enrichment

Dried peptides were redissolved in NETN buffer (50 mM Tris-HCl, 100 mM NaCl, 1 mM EDTA, 0.5% NP-40, pH 8.0) and mixed with 2-hydroxyisobutyryllysine antibody agarose beads (PTM-801 Biolabs) which had been pre-washed three times by NETN buffer, followed by incubation at 4°C overnight with gentle shaking. Beads were washed three times by NETN buffer and twice by ice–cold ddH_2_O to remove unbounded peptides. The bound peptides were eluted from beads by adding 0.1% trifluoroacetic acid (TFA), followed by desalination using C18 ZipTips (Millipore) and vacuum concentration to dry.

### LC-MS/MS Analysis

Enrichment of K_*hib*_ peptides were analyzed using liquid chromatography tandem mass spectrometry (LC-MS/MS) according to the previous method (Baker and Panisko, 2011; Xue et al., 2018) with some modifications. Briefly, peptides were dissolved in solvent A (0.1% formic acid in ddH_2_O) and loaded onto a reversed-phase precolumn (Acclaim PepMap 100 C_18_ column, 2 μm, 75 μm × 20 mm, Thermo Fisher Scientific) after centrifugation at top speed for 5 min. Peptides separation was performed using a reversed-phase analytical column (Acclaim PepMap RSLC C_18_ column, 2 μm, 75 μm × 500 mm, Thermo Fisher Scientific) at 40°C and gradient elution on an Ultimate RSLCnano 3000 system (Thermo Fisher Scientific). Flow rate was 250 μl and the gradient was as follows: 2–10% solvent B (0.1% formic acid in 80% acetonitrile) for 6 min, 10–20% for 45 min, 20–80% for 7 min and then held at 80% for 4 min. Peptides were detected by MS/MS using Q Exactive HFX (Thermo Fisher Scientific) coupled online to LC at a resolution of 60,000. Peptides were selected for MS/MS using a normalized collision energy (NCE) setting of 26%. Ion fragments were detected in orbitrap at a resolution of 30,000. Electrospray voltage was setting to 2.0 kV and m/z scans range was 350–1,800 for MS scans.

### Database Search

Quantitative proteomics software package MaxQuant (v.1.5.2.8) was used for MS/MS raw data analysis (Cox and Mann, 2008; Tyanova et al., 2016). The tandem mass spectra collected were searched against EnzemblFungi *B. cinerea* B05.10 database (ASM83294v1; 11707 coding genes) concatenated with reverse decoy database. Various parameters were set as follows: Trypsin/P was specifically designated as cleavage enzyme and up to four missing cleavage, five modifications per peptide and five charges were allowed. The maximum permissible mass errors of precursor and fragment ions are set at 10 ppm and 0.02 Da, respectively. Carbamido methylation on Cysteine residue was specified as fixed modification while oxidation of methionine residue and 2-hydroxyisobutyrylation both on lysine residue and protein N-terminus were designated as variable modifications. False discovery rate (FDR) thresholds for protein, peptide and modification sites were designated at 0.01 (Elias and Gygi, 2007) and minimal peptide length was designated as 7. K_*hib*_ site localization probability was set to greater than 0.75.

### Bioinformatics Analysis

For motif enrichment, Motif-x platform^1^ (Chou and Schwartz, 2011) was used for analysis of model sequences which were constituted with amino acids in specific positions of modifier-21-mers with 10 amino acid residues upstream and downstream of t K_*hib*_ sites in all protein sequences. Database protein sequences were used as background database parameter and other parameters were set as default (Zhu et al., 2016).

In order to characterize K_*hib*_ proteins identified in the data, function, and characteristics of these proteins were annotated in detail from the perspective of gene theory (GO), protein domain, Kyoto Encyclopedia of Genes and Genomes (KEGG) pathway and subcellular localization. UniProt-GOA database^2^ and platform IterPro^3^ (McDowall and Hunter, 2011) were selected for protein domain and GO term annotation analysis. Based on GO term annotation, proteins were classified into three categories, including biological process, cellular compartment, and molecular function (Reference Genome Group of the Gene Ontology Consortium, 2009). Platform Automatic Annotation Server (KAAS)^4^ and KEGG Mapper^5^ were selected for KEGG annotation analysis. Software Wolfpsort (v.0.2) was selected for subcellular localization analysis. The analysis was performed according to previous studies (Horton et al., 2007; Moriya et al., 2007) and the default setting was used for other parameters. In addition, functional enrichment analyses were performed by the tool of DAVID bioinformatics resources. A two-tailed Fisher’s exact test was employed to test the enrichment of identified 2-hydroxyisobutyrylated proteins against background all proteins of *B. cinerea*. Correction for multiple hypothesis testing was performed using standard FDR control methods. A corrected *p*-value below 0.05 was considered significant for all the enrichment analysis (Huang et al., 2007).

For further hierarchical clustering based on different protein functional classification, enriched substrate categories were filtered for those categories which were at least enriched in one of the clusters with *p*-value below 0.05. Filtered *p*-value matrix was transformed to *z*-scores which were then clustered by one-way hierarchical clustering (Euclidean distance, average linkage clustering) in Genesis. Finally, Cluster membership were visualized by a heat map using the “heatmap.2” function from the “gplots” R-package.^6^

For protein-protein interaction networks (PPI) analysis, search tool for Retrieval of Interacting Genes/Proteins (STRING) database^7^ was employed for functional interaction annotations of all identified 2-hydroxyisobutyrylated proteins by calculating their confidence score. Threshold scores of high-confidence interactions (score > 7) between 2-hydroxyisobutyrylated proteins and high confidence interactions (with score > 0.7) in STRING database were setting at fetched for the analysis. Software Cytoscape was employed for interaction network visualization processing.

## Results

### Identification of Lysine 2-Hydroxyisobutyrylated Proteins in *B. cinerea*

To identify K_*hib*_ sites in *B. cinerea* hyphae, affinity enrichment and high-resolution liquid chromatography–tandem mass spectrometry (LC-MS/MS) methods were used for proteome-wide analysis following the standard workflow (Supplementary Figure 1A). Briefly, total proteins were extracted from the hyphae and digested by trypsin, followed by peptide affinity enrichment using the 2-hydroxyisobutyryl lysine-specific antibody. Then, the peptides containing K_*hib*_ modification were fractionated and loaded on a LC-MS/MS device for identification. Finally, the raw data were analyzed by related software and platforms. Three biological repeats were performed under the same conditions, resulting in 6,551 K_*hib*_ sites on 1,383 proteins, 6,805 K_*hib*_ sites on 1,421 proteins and 6,687 K_*hib*_ sites on 1,398 proteins (Supplementary Tables 1–3). In total, 8,020 K_*hib*_ sites were obtained, in which 5,398 K_*hib*_ sites on 1,181 proteins were found in all three repeats, indicating good repeatability (Supplementary Figure 2 and Supplementary Table 4). Up to now, 14,262 gene transcripts have been annotated in *B. cinerea* B05.10 genome^8^, indicating that the identified 2-hydroxyisobutyrylome contained about 8.3% proteins of the proteome (1,181/14,262). Two representative MS/MS spectra of K_*hib*_ peptides were presented in Supplementary Figures 1B,C. In recent years, K_*hib*_ modification has been reported and shown to play significant roles in several species. A large number of K_*hib*_ sites were identified in our study, indicating that K_*hib*_ modification of proteins is a widespread PTM and may play important roles in the *B. cinerea* cellular process.

### Analysis of K_*hib*_ Site Motifs

**FIGURE 1:**
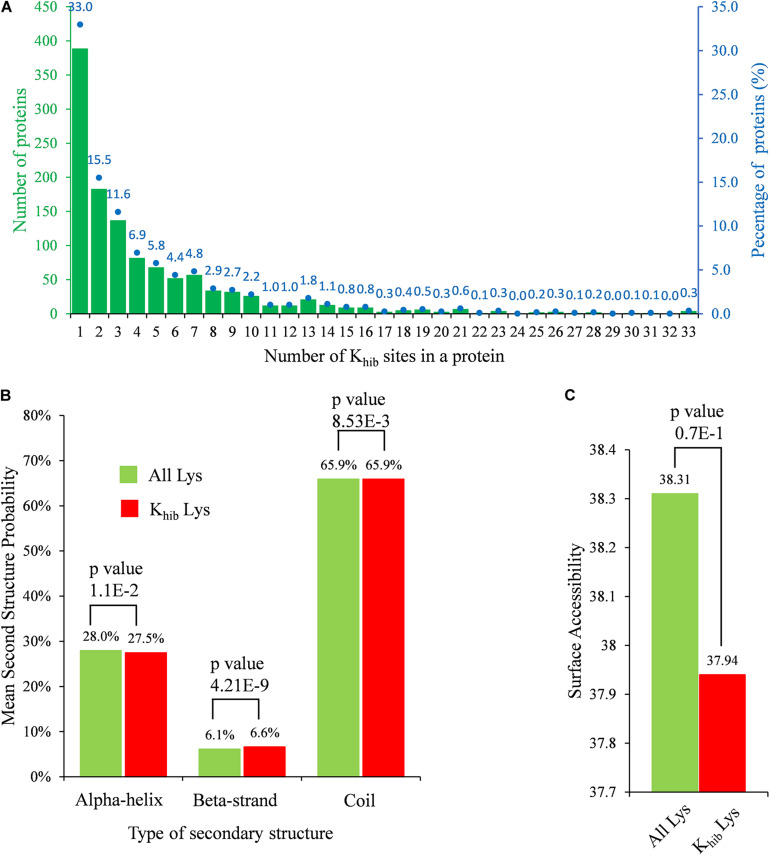
Properties of identified K_*hib*_ sites in *B. cinerea*. **(A)** Distribution of K_*hib*_ sites in identified 2-hydroxyisobutyrylated proteins. The left ordinate indicates the number of proteins with the indicating number of K_*hib*_ sites (*x*-axis), the right ordinate blue dots and number (above the histogram) represents percentages of total identified proteins. **(B)** Probabilities of K_*hib*_ sites in different protein secondary structures. **(C)** Predicted surface accessibility of all lysine residues and in *B. cinerea*. *p*-value < 0.05 is regarded to be significant.

To investigate the distribution of K_*hib*_ sites in *B. cinerea*, the number of modified sites in each identified protein was counted. The K_*hib*_ sites in a protein were distributed from 1 site to more than 30 sites, of which over 60% of the identified 1,181 proteins carried 1–3 K_*hib*_ sites, while approximately 10.2% of the proteins carried more than 10 K_*hib*_ sites (Figure 1A).

The secondary structure analysis was performed using NetSurfP to determine the preferred structure of the K_*hib*_ site in proteins. In *B. cinerea*, both lysine and K_*hib*_ were mostly located at the coil region, with a percentage of 65.9% (Figure 1B), while K_*hib*_ tended to occur more frequently at the beta-strand region than at the alpha-helix region compared to unmodified lysine residues (*p*-value = 4.21E-09 and 1.10E-02 for beta-strand and alpha-helix, respectively). In addition, 2-hydroxyisobutyrylated sites were less surface assembled than unmodified lysine residues, but the difference might not be significant because of a high *p*-value of 0.7 (> 0.05) (Figure 1C). There were no obvious differences in the preference of secondary structure by K_*hib*_ modification, which might be because this modification occurs in all kinds of proteins in *B. cinerea*.

**FIGURE 2:**
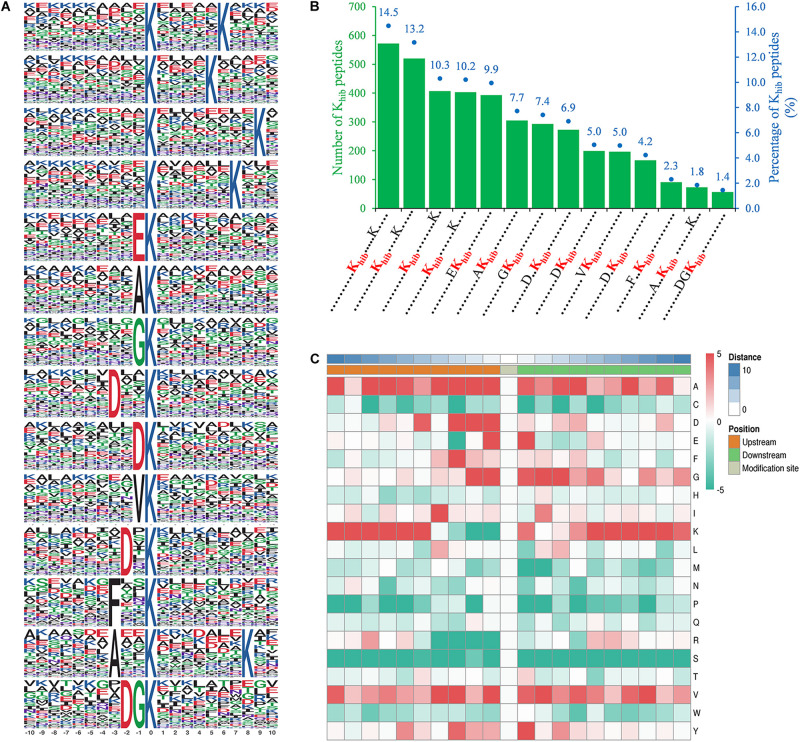
Properties of identified K_*hib*_ peptides in *B. cinerea*. **(A)** Peptide motifs with conserved residues around K_*hib*_ sites. **(B)** Frequency of identified K_*hib*_ peptides in each conserved motif. The left ordinate indicates number of the indicating conserved motif in *x*-axis, the right ordinate with blue dots and number (above the histogram) represents percentages of total conserved motifs. K_*hib*_ in red represents 2-hydroxyisobutyrylated lysine residue and each dot represents an amino acid residue. **(C)** Heat map of the indicating amino acid residues (right letter) around K_*hib*_ sites in identified peptides. The middle represents K_*hib*_ sites, left (croci), and right (green) grids represent upstream and downstream residues of K_*hib*_ sites, respectively. The darker the red, the higher the frequency and the deeper the green, the lower the frequency.

Motif-x software was used to detect the specific amino acid sequence motifs around K_*hib*_ sites. A total of 14 conserved motifs were identified for 10 amino acids upstream and downstream of K_*hib*_ sites (-10 K_*hib*_ +10) in 3,950 peptides, accounting for 73.2% of the total identified peptides (Figure 2A). The amino acids around these K_*hib*_ sites showed a diverse distribution in *B. cinerea*, while another lysine residue downstream of a K_*hib*_ site (+5 to +9) seemed to have an extreme preference for the 2-hydroxyisobutyryl modification, and this occurred in a total of 50% of the K_*hib*_ sites (Figure 2B). In addition, five conserved motifs, [EK_*hib*_], [DxxK_*hib*_], [DK_*hib*_], [DxK_*hib*_], and [DGK_*hib*_] (K_*hib*_ indicates the 2-hydroxyisobutyrylated lysine, and x indicates a random amino acid residue), were identified in the 2-hydroxyisobutyrylsome of *B. cinerea* with a total percentage of 27.4% (Figures 2A,B). These five conserved motifs have been identified in other species (Meng et al., 2017; Yu et al., 2017; Huang et al., 2018), indicating that an amino acid with a negative charge (D or E) seemed more suitable for the 2-hydroxyisobutyryl modification of a downstream lysine. Furthermore, the frequency of amino acids flanking the K_*hib*_ site is shown in a heatmap (Figure 2C). In addition to a downstream unmodified lysine residue, an unmodified lysine residue also occurred most frequently -10 to -5 residues upstream from the modification sites. Several small amino acids with short side chains, alanine (A), glycine (G), and valine (V), were more present around the modified K sites, while some polar amino acids, serine (S), cysteine (C) and arginine (R), and proline (P), were less present around the K_*hib*_ sites (Figures 2B,C).

### Conserved Analysis of K_*hib*_ Proteins

**FIGURE 3:**
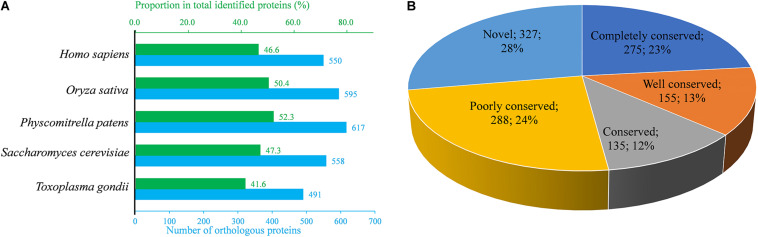
Conservation analysis of identified K_*hib*_ proteins in *B. cinerea* compared with several species. **(A)** Orthologs analysis of identified K_*hib*_ proteins in *Homo sapiens*, *Oryza sativa*, *Physcomitrella patens*, *Saccharomyces cerevisiae* and *Toxoplasma gondii* with their reported 2-hydroxyisobutyrylomes. The under horizontal axis (blue) indicates number of orthologs in the indicating species while the top horizontal axis (green) represents the proportion in total identified K_*hib*_ proteins. **(B)** A pie chart of conserved K_*hib*_ proteins in five species. Completely conserved group means that the identified K_*hib*_ protein has five orthologs in the above five species, while Well conserved group means four orthologs, Conserved group means three orthologs, Poorly conserved group means one or two orthologs and Novel group means zero orthologs.

To understand the evolutionary conservation of K_*hib*_ proteins in different species, identified K_*hib*_ protein sequences of *B. cinerea* were compared against K_*hib*_ protein sequences from five other species, including *Homo sapiens*, *Oryza sativa* subsp. Japonica, *Physcomitrella patens*, *Saccharomyces cerevisiae*, and *Toxoplasma gondii* (Dai et al., 2014; Huang et al., 2017; Meng et al., 2017; Yu et al., 2017; Yin et al., 2019), using BLASTP. Among the 1,181 identified proteins of *B. cinerea*, the number of orthologous proteins of *H. sapiens*, *O. sativa* subsp. Japonica, *P. patens*, *S. cerevisiae* and *T. gondii* were 550, 595, 617, 558, and 491, respectively (Figure 3A and Supplementary Table 5). The proportions of orthologous proteins in the two plant species (*O. sativa* subsp. Japonica and *P. patens*) were more than 50% (595/1,181 and 617/1,181, respectively). The proportions of orthologous proteins in *H. sapiens* and *S. cerevisiae* were 46.6% (550/1,181) and 47.3% (558/1,181), respectively, while in *T. gondii*, the proportion was relatively less (Figure 3A). Among the 1,181 identified proteins, 275 (accounting for 23.3%) proteins were found in all five species and classified as completely conserved proteins; 155 (accounting for 13.1%) proteins were found in four of the five species and classified as well conserved proteins; 135 (accounting for 11.4%) proteins were found in three of the five species and classified as conserved proteins, 288 (accounting for 24.4%) proteins were found in one or two of the five species and classified as poorly conserved proteins, and 327 (accounting for 27.7%) proteins did not have an ortholog in any of the five species and were classified as novel proteins (Figure 3B and Supplementary Table 5).

### Functional Annotation and Subcellular Localization of K_*hib*_ Proteins

**FIGURE 4:**
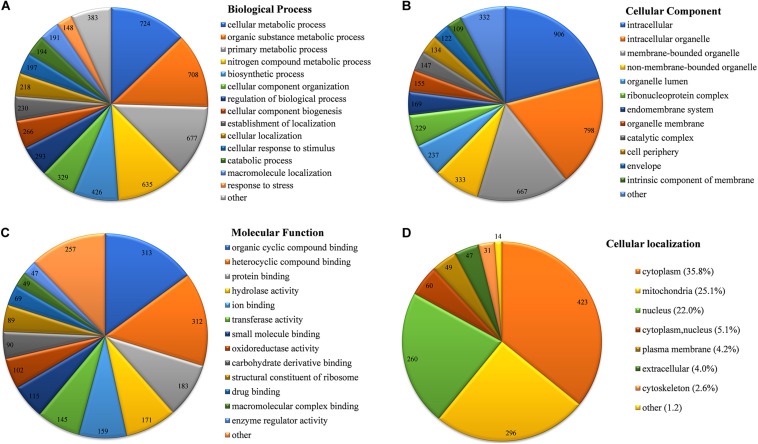
Functional classification of identified K_*hib*_ proteins in *B. cinerea*. **(A)** Classification of K_*hib*_ proteins based on biological process. **(B)** Classification of K_*hib*_ proteins based on cellular component. **(C)** Classification of K_*hib*_ proteins based on molecular function. **(D)** Subcellular localization of identified K_*hib*_ proteins in *B. cinerea*. Values on the pie chart represent protein number classified in the indicating terms.

Based on a Gene Ontology (GO) term classification analysis, the identified K_*hib*_ proteins in *B. cinerea* were classified into three categories, biological process, cell composition, and molecular function, which contained several GO terms (Figure 4 and Supplementary Table 6). In the category of biological process, the top four GO terms with the largest number of proteins were “cellular metabolic process,” “organic substance metabolic process,” “primary metabolic process” and “nitrogen compound metabolic process,” containing 724, 708, 677, and 635 identified proteins, respectively (Figure 4A). Each of the four terms contained more than half of the total identified proteins (1,181), indicating that most identified K_*hib*_ proteins were associated with metabolism. In the category of cell composition, the top two GO terms with the largest number of proteins were “intracellular” and “intracellular organelle,” containing 906 and 798 identified proteins, respectively (Figure 4B), indicating that most identified K_*hib*_ proteins were distributed in the matrix of cells or in organelles. In the category of molecular function, the top three GO terms with the largest number of proteins were “organic cyclic compound binding,” “heterocyclic compound binding,” and “protein binding,” containing 313, 312, and 183 identified proteins, respectively (Figure 4C). To further study the functional classification, Clusters of Orthologous Groups/euKaryotic Orthologous Groups (COG/KOG) database alignment was performed for the identified K_*hib*_ proteins. This analysis identified 1,094 proteins (accounting for 92.7% of the total identified K_*hib*_ proteins) and divided them into 23 COG/KOG categories, with “translation, ribosomal structure and biogenesis” (176), “PTM, protein turnover, chaperones” (141) and “energy production and conversion” (99) being the three most highly represented categories (Supplementary Figure 3 and Supplementary Table 7).

Subcellular localization of the identified K_*hib*_ proteins in *B. cinerea* was analyzed by WoLF PSORT software. The K_*hib*_ proteins were mainly localized in the cytoplasm, mitochondria, and nucleus, containing 35.8, 25.1 and 22.0% of the total identified proteins, respectively (Figure 4D and Supplementary Table 8). In addition, 5.1% of the total identified proteins showed both cytoplasmic and nuclear localization. Other small amounts of proteins were localized in the plasma membrane (4.2%), extracellular space (4.0%), and cytoskeleton (2.6%), indicating that K_*hib*_ proteins in *B. cinerea* are preferred to be of intracellular localization.

### Functional Enrichment Analysis of K_*hib*_ Proteins

**FIGURE 5:**
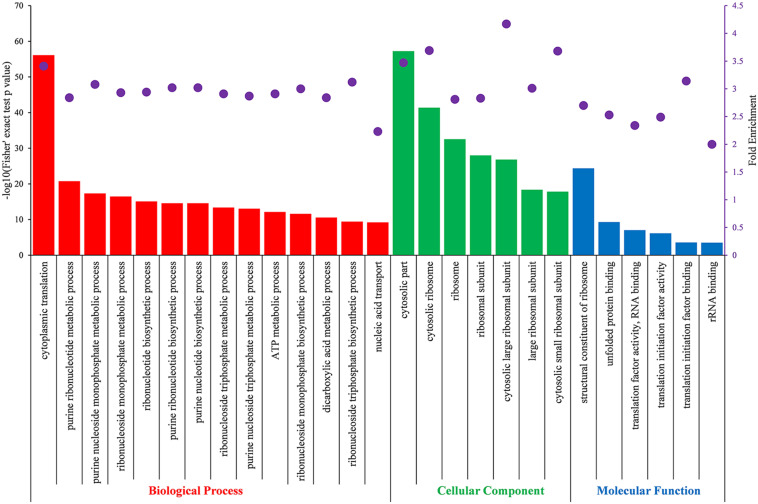
Partial significantly enriched GO terms of identified K_*hib*_ proteins based on biological process (red), cellular component (green) and molecular function (blue) with a Fisher’s exact test *p* < 0.05 and fold enrichment value > 2.0. The left ordinate indicates the value of -log10 (Fisher’s test *p*-value) (black) of the indicating terms in *x*-axis. The right ordinate and the purple dots represent fold enrichment of the indicating terms in *x*-axis.

To further understand the preferred protein types, metabolic pathways and protein domains of K_*hib*_ proteins in *B. cinerea*, GO, Kyoto Encyclopedia of Genes and Genomes (KEGG) and domain enrichment analyses were performed for the identified proteins. Enriched GO terms with a Fisher’s exact test *p*-value < 0.05 were listed in Supplementary Table 9, and enriched GO terms with fold enrichment value > 2 were shown in Figure 5. The results revealed that K_*hib*_ proteins in *B. cinerea* were involved in multiple pivotal metabolic processes or pathways. Enrichment analysis of GO biological processes demonstrated that the identified K_*hib*_ proteins were associated with cytoplasmic translation and substance metabolism and biosynthesis, especially with purine ribonucleotide and purine nucleoside metabolic and biosynthetic processes, which are widely involved in energy supply, metabolic regulation and coenzyme composition. Enrichment analysis of GO cellular components demonstrated that the identified K_*hib*_ proteins were mainly involved in ribosome composition and seemed to be closely related to protein synthesis. Enrichment analysis of GO molecular functions revealed that the identified K_*hib*_ proteins played key functions in many aspects of protein expression, including structural constituents of ribosomes, translation, initiation, mRNA and rRNA binding, protein folding, etc.

**FIGURE 6:**
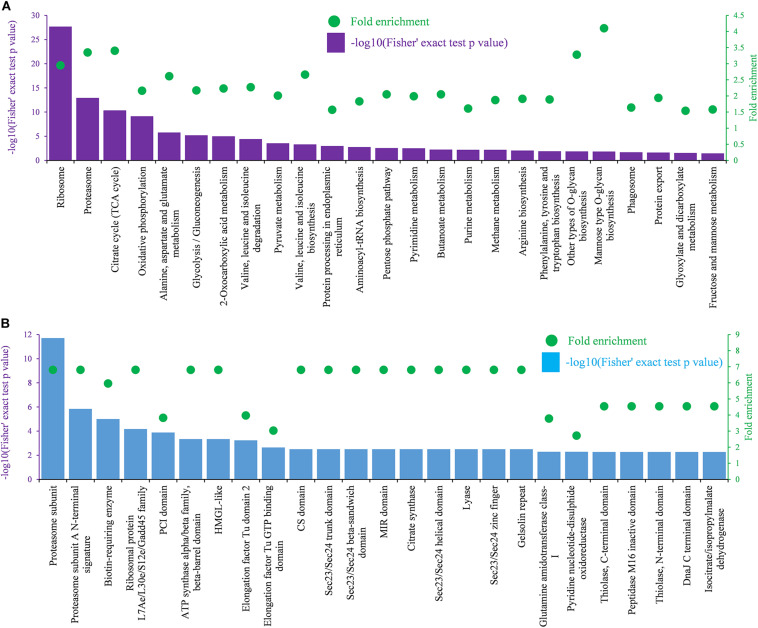
KEGG and domain enrichment analysis of identified K_*hib*_ proteins. **(A)** Enrichment analysis based on KEGG pathways. **(B)** Enrichment analysis based on functional domains. The left ordinate indicates value of -log10 (Fisher’s test *p*-value) (black) of the indicating terms in *x*-axis. The right ordinate and the green dots represent the fold enrichment of the indicating terms in *x*-axis.

Metabolic pathway enrichment analysis using the KEGG pathway annotation database revealed that the identified K_*hib*_ proteins were enriched in 25 pathways with a Fisher’s exact test *p*-value < 0.05 and fold enrichment value > 1.5 (Figure 6A). In these enriched pathways, the two highest enriched pathways were the ribosome pathway (map03010) (Supplementary Figure 4A) and the proteasome pathway (map03050), which were associated with protein synthesis and degradation. In addition, several other enriched pathways were also related to protein synthesis and processing in cells, including protein processing in the endoplasmic reticulum (map04141), aminoacyl-tRNA biosynthesis (map00970), protein export (map03060), RNA transport (map03013), and amino acid metabolism (map00250, map00290, map00220, and map00400). In addition, several enriched pathways were related to energy metabolism with adenosine triphosphate (ATP) production, including the citrate cycle (TCA cycle) (map00020) (Supplementary Figure 4B), oxidative phosphorylation pathway (map00190), and glycolysis/gluconeogenesis pathway (map00010) (Figure 6A). Protein domain enrichment analysis showed that the identified K_*hib*_ proteins were enriched in 25 domain families with a Fisher’s exact test *p*-value < 0.05 and fold enrichment value > 2 (Figure 6B). Two top enriched protein families were related to the proteasome subunit, in which all proteins had a K_*hib*_ modification. In addition, proteins containing those domains, such as ribosomal protein L7Ae/L30e/S12e/Gadd45 family, ATP synthase alpha/beta family, beta-barrel domain, biotin-requiring enzyme, etc., showed a higher tendency to be K_*hib*_-modified (Figure 6B).

### Protein–Protein Interaction (PPI) Network Analysis of K_*hib*_ Proteins

**FIGURE 7:**
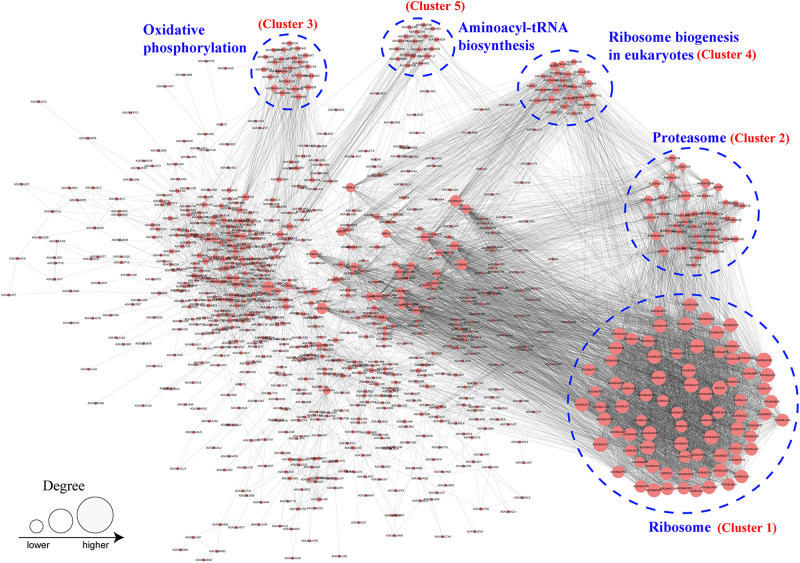
Protein–protein interaction network analysis of identified K_*hib*_ proteins in *B. cinerea*. The top five cluster with highly interconnected were associated with ribosome, proteosome, oxidative phosphorylation, ribosome biogenesis in eukaryotes and aminoacyl-tRNA biosynthesis and indicated by blue dotted circle. Size of the circle indicates number of K_*hib*_ sites in each proteins.

PPI network analysis is helpful to clarify the relationship between different protein and important for investigating the function of proteins in molecular processes (Szklarczyk et al., 2019). To investigate the function of K_*hib*_ proteins in *B. cinerea*, a PPI network was established using the STRING database. In total, 895 K_*hib*_ proteins were mapped to the PPI database, and 506 of them were retrieved to 47 clusters that were highly interconnected (Figure 7 and Supplementary Table 10). The top five clusters (clusters 1–5) were associated with ribosomes, proteosomes, oxidative phosphorylation, ribosome biogenesis in eukaryotes and aminoacyl-tRNA biosynthesis, and included 81, 42, 20, 22, and 12 proteins, respectively (Figure 7 and Supplementary Table 10). The results revealed that the K_*hib*_ proteins formed complicated interaction networks through direct or indirect physiological cooperation and coordination, which may be significant to exert their function in *B. cinerea*.

### Functional Analysis of K_*hib*_ Proteins Involved in the Pathogenicity of *B. cinerea*

**FIGURE 8:**
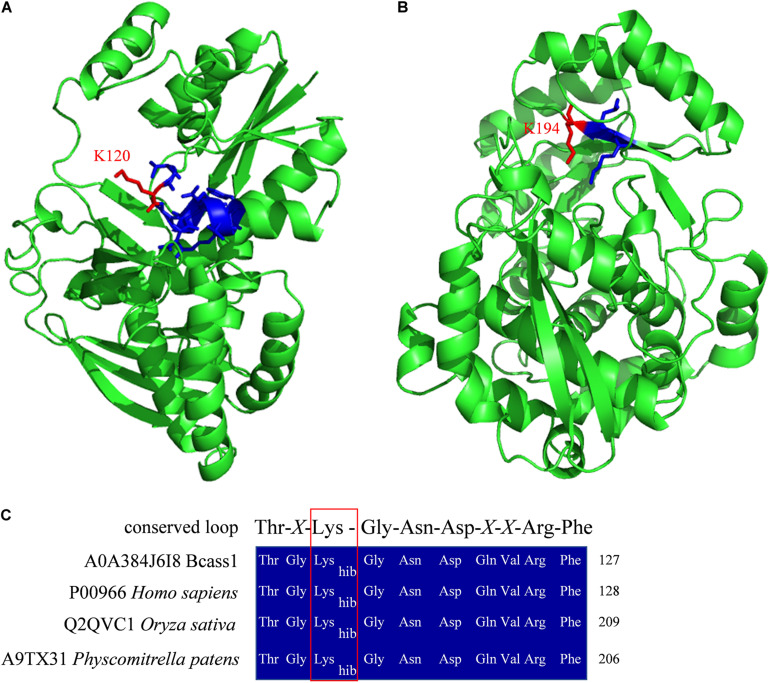
Protein structure homolog modeling and sequence alignment. Three-dimensional structure models of Bcass1 **(A)** and Bclgd1 **(B)**. Two protein structure models were modeled by SWISS-MODEL platform (Waterhouse et al., 2018) based on human argininosuccinate synthetase structure (PDB 2nz2) and human reverse thymidylate synthase structure (PDB 4a35), respectively. Structures were shown in green cartoon. Functional domains were shown in blue sticks and the K_*hib*_ sites identified in our study were shown in red sticks. **(C)** Sequence alignment of the conserved loop of Thr-*X*-Lys-Gly-Asn-Asp-*X*-*X*-Arg-Phe in *B. cinerea*, human, rice, and *Physcomitrella patens*. The red box indicated the 2-hydroxyisobutyrylated lysine sites.

*B. cinerea* is one of the most destructive plant pathogens, can infect more than 200 plants and is thus a model generalist pathogen for studying the interactions between plant hosts and fungal pathogens (Soltis et al., 2019; Xiong et al., 2019). To infect hosts successfully, pathogens, such as *B. cinerea*, employs multiple strategies based on quantitative genetic architectures, including numerous extracellular enzymes, proteins, metabolite and battling with hosts in metabolic levels (Kliebenstein et al., 2005; Nakajima and Akutsu, 2014; Corwin and Kliebenstein, 2017; Zhang et al., 2019). In recent years, epigenetic regulation was also reported to be involved in the regulation of pathogenicity of pathogens (Dubey and Jeon, 2017; Izbiańska et al., 2019). In this study, we found that several identified K_*hib*_ proteins had been reported to function in the pathogenicity of *B. cinerea* (Table 1 and Supplementary Table 11), indicating that as a recently identified protein PTM, K_*hib*_ of proteins may also be involved in the regulation of pathogenicity. The K_*hib*_ proteins involved in the pathogenicity of *B. cinerea* were divided into five categories according to their biological functions, including substance synthesis and metabolism, redox and autophagy, kinase, protease, and other functions (Table 1). Moreover, several K_*hib*_ sites were located in or close to the functional domains in these proteins. For example, K120 was identified to be 2-hydroxyisobutyrylated in argininosuccinate synthase (Bcass1) (Supplementary Table 11), and this site is located in the predicted conserved loop of Thr^118^-*X*-Lys^120^-Gly^121^-Asn^122^-Asp^123^-*X*-*X*-Arg^126^-Phe^127^ (Figure 8A) which interacts with the substrates in human and *Thermus thermophilus* (Goto et al., 2002; Karlberg et al., 2008). Five K_*hib*_ sites (K157, K173, K177, K194, K262) were identified in *B. cinerea* L-galactonate dehydratase (Bclgd1), among which the K194 is the second K locating in the predicted K × K motif (Figure 8B) which had been reported to function for base-catalyzed proton abstraction in human (Wichelecki et al., 2014). Interestingly, the lysine 2-hydroxyisobutyrylation was conservative in the conserved loop of Thr-*X*-Lys-Gly-Asn-Asp-*X*-*X*-Arg-Phe in human, rice and *Physcomitrella patens* (Meng et al., 2017; Yu et al., 2017; Huang et al., 2018; Figure 8C). These results indicate that K_*hib*_ may play a regulatory role by affecting protein key functional domains.

**TABLE 1:** List of identified K_*hi*_b proteins involved in pathogenicity of *B. cinerea.*

Category	Name	Functions	References
Substance synthesis and metabolism	Bcass1	Argininosuccinate synthase	Patel et al., 2010
	Bclgd1	Galactonate dehydratase, D-galacturonic acid catabolic pathway	Zhang and van Kan, 2013
	Bclga1	Galactonate aldolase, D-galacturonic acid catabolic pathway	Zhang and van Kan, 2013
	Bcgar1	Galacturonate reductase, D-galacturonic acid catabolic pathway	Zhang and van Kan, 2013
	BcCHSVI	Chitin synthase	Cui et al., 2013
	Bcpck1	Phosphoenolpyruvate carboxykinase, gluconeogenesis	Liu et al., 2018
	Bcbrn1	Tetrahydroxynaphthalene reductases	Zhang et al., 2015
	Bcbrn2	Tetrahydroxynaphthalene reductases	Schumacher, 2016
	Bcscd1	Scytalone dehydratase	Schumacher, 2016
	BcBOA1	Key enzyme for botcinic acid biosynthesis	Zhang et al., 2019; Soltis et al., 2020
	BOA6	Key enzyme for botcinic acid biosynthesis	Dalmais et al., 2011
	Bccpr1	Cytochrome P450 oxidoreductase	Siewers et al., 2004
Redox and autophagy	Bcsod1	Cu-Zn-superoxide dismutase	López-Cruz et al., 2017
	Bcglr1	Glutathione reductase, cellular redox system	Viefhues et al., 2014
	Bctrr1	Thioredoxin reductase, cellular redox system	Viefhues et al., 2014
	Bcatg8	Autophagy pathway	Ren et al., 2018a
	Bcatg3	Autophagy pathway, ubiquitin-like activating enzyme E2	Ren et al., 2018b
Kinase	Bccla4	PAK kinase, effector of Rac	Minz-Dub and Sharon, 2017
	Bcmkk1	MAPK kinase, suppresses oxalic acid biosynthesis	Yin et al., 2018
	Bos5	Mitogen-activated protein kinase	Yan et al., 2010
	Bcsak1	Mitogen-activated protein kinase	Segmüller et al., 2007
	Bmp1	Mitogen-activated protein kinase	Zheng et al., 2000
	Bmp3	Mitogen-activated protein kinase	Rui and Hahn, 2007
Protease	Bcacp1	Proteases, G1 family	Rolland et al., 2009
	Bcser2	Subtilisin-like protease	Liu et al., 2020
	Bcser1	Subtilisin-like protease	Liu et al., 2020
Others	Bcspl1	Cerato-platanin family protein; SAR inducer for host	Frías et al., 2011, 2013
	Bcptc3	Type 2C protein phosphatases	Yang et al., 2013
	Bcpdi1	ER protein, interaction partner of the NoxA complex	Marschall and Tudzynski, 2017
	Bccdc42	Small GTPase	Kokkelink et al., 2011
	Bcpg1	Endopolygalacturonase	ten Have et al., 1998
	Bcsec31	Vesicle transport	Zhang et al., 2016
	BcactA	Actin	Li et al., 2020
	BcP1	Cyclophilin A	Viaud et al., 2003
	CND6	ATP citrate lyase	Viaud et al., 2003
	CND16	ATP citrate lyase	Viaud et al., 2003
	CND9	Mannitol-1-phosphate dehydrogenase	Viaud et al., 2003

## Discussion

K_*hib*_ is a protein PTM recently found in histones and non-histone proteins in several species. In this study, K_*hib*_ proteins in *B. cinerea* were investigated by proteome-wide analysis. A total of 5,398 K_*hib*_ sites on 1,181 proteins were identified from all three biological repeats, accounting for approximately 10% of the *B. cinerea* proteome, which is much more than the acetylome in *B. cinerea* (Lv et al., 2016), indicating that K_*hib*_ is a slightly more abundant PTM in *B. cinerea*. Most proteins contain a few K_*hib*_ sites (no more than three sites, accounting for over 60% of proteins), and the modified sites are distributed in different protein secondary structures (Figure 1), indicating that K_*hib*_ modification occurs in different types of proteins. Analysis of amino acid sequence motifs around the K_*hib*_ sites showed that the modification preferred to occur near negatively charged or small amino acids (Figure 2). Similar preferences were found in the recently identified K_*hib*_ modification proteome. For examples, in the 2-hydroxyisobutyrylome of developing rice seeds, the motifs, [EK_*hib*_], [DxxK_*hib*_], [DK_*hib*_], and [DxK_*hib*_], have been identified as enriched motifs, and the negatively charged side chain amino acids, D and E, also showed a strong bias around the modified lysine residues (Meng et al., 2017). In the 2-hydroxyisobutyrylome of HeLa cells, the negatively charged amino acids (D and E) were enriched at both -1 and +1 positions of K_*hib*_ (Huang et al., 2018). In the 2-hydroxyisobutyrylome of *Physcomitrella patens*, A heatmap analysis showed that the amino acid D and E were overrepresented in the near upstream position of K_*hib*_ site (Yu et al., 2017). All the results appear to reveal that the position of lysine in the amino acid sequence plays a decisive role in its K_*hib*_ modification.

Protein conservation analysis showed that the K_*hib*_ modification proteome of *B. cinerea* contains both conserved and newly identified proteins when compared with the K_*hib*_ proteins in the above eukaryotes (Figure 3), suggesting that K_*hib*_ modification may be involved in different cellular processes and regulation pathways in different species. Functional classification analysis showed that the identified K_*hib*_ proteins are distributed in almost all parts of the cell and play functions in various aspects, including the composition of cell structures, metabolism of substances, generation of energy, expression and function of proteins, transduction and regulation of signals (Figure 4). From the results of GO and KEGG enrichment analyses (Figures 5, 6), we can see that the identified K_*hib*_ proteins in *B. cinerea* were highly enriched in the ribosome, cellular machinery of protein synthesis (Emmott et al., 2019), translation initiation, mRNA and rRNA binding, protein folding, and the proteasome pathway, which are closely related to protein synthesis or degradation. In addition, PPI network analysis of the identified K_*hib*_ proteins showed that the cluster ribosome and proteasome were the most interconnected (Figure 7). These results suggest that K_*hib*_ modification may play important roles in protein expression and degradation in cells.

From the above results, K_*hib*_ proteins play an indispensable role in maintaining the normal growth, development, and metabolism of *B. cinerea*. Significantly, beyond that, many identified K_*hib*_ proteins have been declared to be associated with regulating the pathogenicity in *B. cinerea* (Table 1, Figure 8, and Supplementary Table 11). For example, the D-galacturonic acid catabolic pathway consists of three catalytic steps: non-homologous galacturonate reductase, galactonate dehydratase and 2-keto-3-deoxy-L-galactonate aldolase catalyzed by Bcgar1, Bcgar2, Bclgd1, and Bclga1, and defects in each step of the pathway showed reduced virulence (Zhang and van Kan, 2013). Dihydroxynaphthalene (DHN) melanin is the major component of the extracellular matrix of *B. cinerea* and has been reported to function in different life processes, including the invading process of the penetration structures and the longevity of the reproduction structures (Zhang et al., 2015; Schumacher, 2016). In our study, three key enzymes of melanogenesis pathway, tetrahydroxy naphthalene reductases (Bcbrn1 and Bcbrn2) and scytalone dehydratase (Bcscd1), were found to be 2-hydroxyisobutyrylated at multiple sites (Supplementary Table 11). Reactive oxygen species (ROS) play important functions in the cellular redox system and cell autophagy. Bcsod1, Bcglr1, Bctrr1, Bcatg3, and Bcatg8 function as superoxide dismutase, glutathione reductase, thioredoxin reductase, and autophagy-related proteins, and the absence of each of these proteins reduced *B. cinerea* virulence (Viefhues et al., 2014; López-Cruz et al., 2017; Ren et al., 2018a,b). In *B. cinerea*, kinases play an important role in signal transduction pathways and participate in the regulation of pathogenicity (Zheng et al., 2000; Rui and Hahn, 2007; Segmüller et al., 2007; Yan et al., 2010; Minz-Dub and Sharon, 2017; Yin et al., 2018). In our study, several protein kinases, such as Bccla4, Bcmkk1, Bos5, Bcsak1, Bmp1, and Bmp3, were identified as K_*hib*_ proteins (Supplementary Table 11). Some enzymes, such as the protease Bcacp1, subtilisin-like proteases Bcser1 and Bcser2, protein phosphatase Bcptc3, and endopolygalacturonase Bcpg1, are also involved in the pathogenicity of *B. cinerea* and were identified in this study (ten Have et al., 1998; Rolland et al., 2009; Yang et al., 2013; Liu et al., 2020; Table 1 and Supplementary Table 11). In addition, the cerato-platanin family protein Bcspl1 not only is related to the pathogenicity of *B. cinerea* but also can induce host immunity and systemic acquired resistance (Frías et al., 2011, 2013), indicating that the K_*hib*_ modification of *B. cinerea* protein may be associated with the induction of host immunity, which has been reported in other kinds of PTMs (de Vega et al., 2018). Bcptc3 is a type 2C Ser/Thr phosphatase (PP2C) that negatively regulates the phosphorylation of Bcsak1, and both proteins are involved in the regulation of pathogenicity (Segmüller et al., 2007; Yang et al., 2013) and were identified as K_*hib*_ proteins (Table 1), which implies that K_*hib*_ modification may regulate pathogenicity by affecting protein phosphorylation and dephosphorylation.

## Conclusion

In conclusion, as mentioned above, K_*hib*_ proteins are widely involved in the growth, development and pathogenicity of *B. cinerea*, and to the best of our knowledge, this is the first proteome-wide analysis of K_*hib*_ in the phytopathogenic fungus *B. cinerea.* Our study provides a foundation and protein candidates for further investigations of the roles and mechanisms of K_*hib*_ in regulating the growth and pathogenicity of *B. cinerea*, which will be helpful for facilitating the development of improved pesticides to control this destructive plant pathogen.

## Data Availability Statement

The original contributions presented in the study are included in the article/Supplementary Material, further inquiries can be directed to the corresponding author.

## Author Contributions

WL and ML generated the hypothesis, planned the experiments, and wrote the manuscript. YX and XL performed the experiments. All other authors provided comments on the manuscript.

## Conflict of Interest

The authors declare that the research was conducted in the absence of any commercial or financial relationships that could be construed as a potential conflict of interest.

## Footnotes

1 http://motif-x.med.harvard.edu/motif-x.html

2 http://www.ebi.ac.uk/GOA/

3 http://www.ebi.ac.uk/interpro/

4 https://www.genome.jp/kaas-bin/kaas_main

5 https://www.kegg.jp/kegg/mapper.html

6 https://cran.r-project.org/web/packages/cluster/

7 https://string-db.org/cgi/input.pl

8 https://fungi.ensembl.org/Botrytis_cinerea/Info/Annotation/#assembly
