# Supplementary material for: Proteome-Wide Analysis of Lysine 2-Hydroxyisobutyrylation in the Phytopathogenic Fungus Botrytis cinerea
Source: Front Microbiol. 2020 Nov 27;11:585614. doi: 10.3389/fmicb.2020.585614 (PMC7728723; doi:10.3389/fmicb.2020.585614)
Supplement: Supplementary file 2 [file Data_Sheet_2.pdf]

Supplementary Material

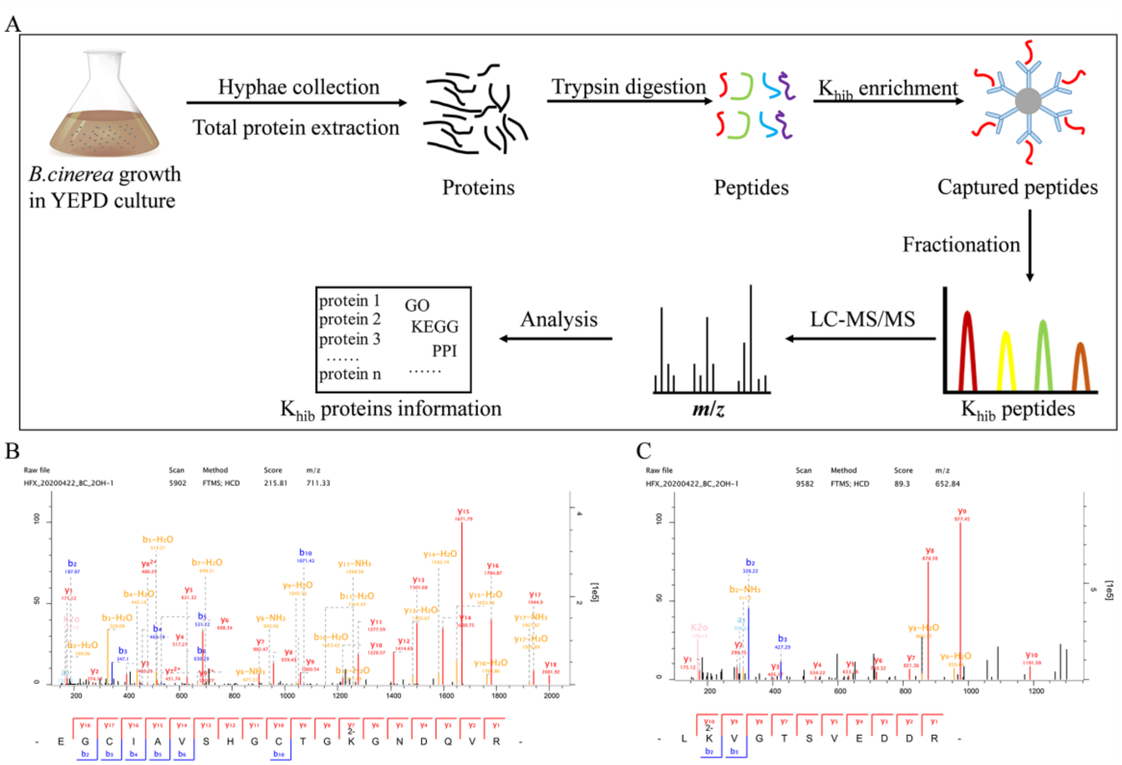

Supplementary Figure 1. Proteome-wide analysis workflow and two representative MS/MS spectra. **(A)** The workflow of lysine 2-hydroxyisobutyrylation (K_{hib}) analysis for total proteins extracted from *Botrytis cinerea* hyphae using affinity enrichment and high-resolution LC-MS/MS methods. **(B)** and **(C)** The MS/MS spectra of peptide EGCIAVSHGCTGK_{hib}GNDQVR with a 2-hydroxyisobutyrylated site at K120 in Bcass1 (A0A384J6I8) and peptide LK_{hib}VGTSVEDDR with a 2-hydroxyisobutyrylated site at K194 in Bclgd1 (A0A384J6S6).

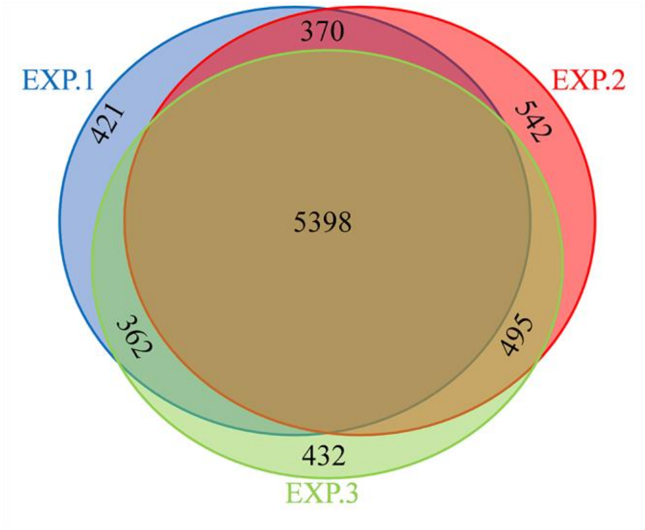

Supplementary Figure 2. Venn diagram of identified K_{hib} sites from three biological repeats. Overlapped areas represent that identified K_{hib} sites were repeated in different biological repeats.

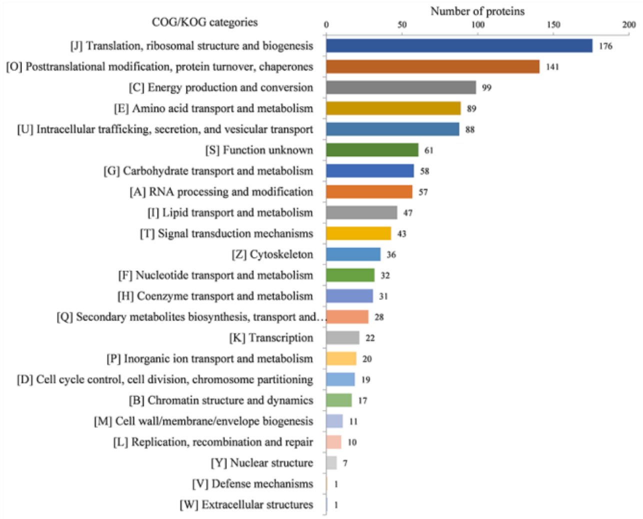

Supplementary Figure 3. Functional classification analysis based on Clusters of Orthologous Groups/EuKaryotic Orthologous Groups (COG/KOG) database. The value side the histogram represents protein number of the indicating terms.

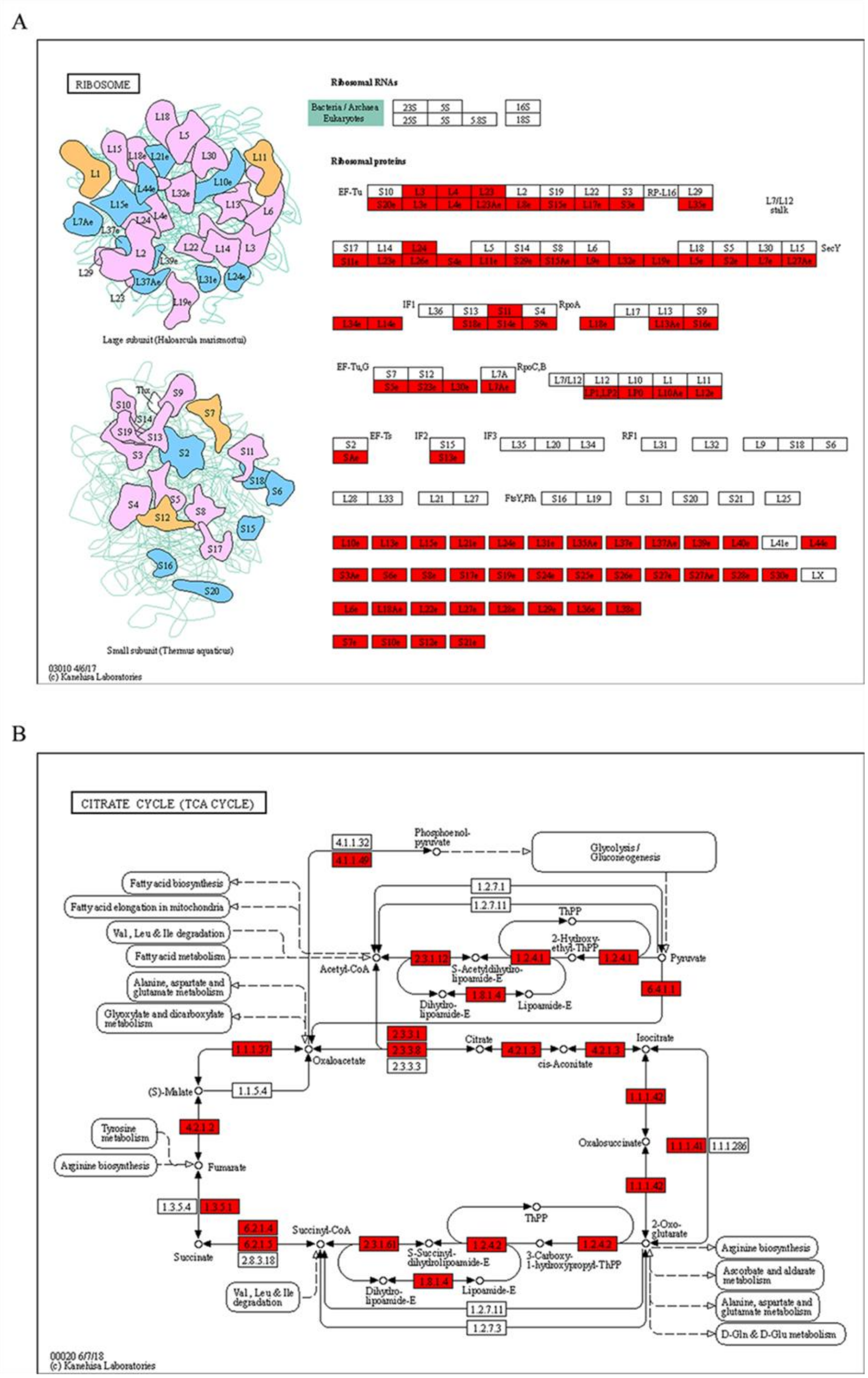

Supplementary Figure 4. Two representatives of enriched KEGG pathways. **(A)** Ribosome pathway (map03010). (B) Citrate cycle (TCA cycle) pathway (map00020). The identified K_{hib} proteins were highlighted in red.

Supplementary Table 1. K_{hib} sites identified in EXP.1 (biological repeat experiment 1).

Supplementary Table 2. K_{hib} sites identified in EXP.2 (biological repeat experiment 2).

Supplementary Table 3. K_{hib} sites identified in EXP.3 (biological repeat experiment 3).

Supplementary Table 4. Total sites of K_{hib} identified in *B.cinerea* hyphae. All of the sites were detected in 3 biological repeats.

Supplementary Table 5. Orthologs of K_{hib} proteins identified from *B.cinerea* in different species. The species include *Homo sapiens*, *Oryza sativa* subsp. japonica, *Physcomitrella patens*, *Saccharomyces cerevisiae* and *Toxoplasma gondii*.

Supplementary Table 6. Information of K_{hib} proteins identified from *B.cinerea* classified into different GO terms.

Supplementary Table 7. Information of K_{hib} proteins identified from *B.cinerea* classified into different COG/KOG terms.

Supplementary Table 8. Subcellular localization details of K_{hib} proteins identified from *B.cinerea*.

Supplementary Table 9. Details of GO enrichment analysis of identified K_{hib} proteins in *B.cinerea*. The GO terms were filtrated with Fisher's exact test P value less than 0.05.

Supplementary Table 10. Details of PPI analysis of identified K_{hib} proteins in *B.cinerea*. The table includes 506 clustered proteins and 389 unclustered prtoeisns.

Supplementary Table 11. Details of 37 identified K_{hib} proteins involved in pathogenicity of *B.cinerea*.
